# On your terms or mine: pigs’ response to imposed gentle tactile contact vs. free form interaction with a familiar human

**DOI:** 10.1038/s41598-024-76451-5

**Published:** 2024-10-24

**Authors:** Suzanne Truong, Oceane Schmitt, Jean-Loup Rault

**Affiliations:** 1 Centre for Animal Nutrition and Welfare, University of Veterinary Medicine Vienna, Veterinaerplatz 1, 1210 Vienna, Austria; 2 Institute of Animal Breeding and Genetics, Faculty of Agricultural Sciences, Nutritional Sciences, and Environmental Management, University of Giessen, Giessen, Germany

**Keywords:** Agency, Expectancy violation, Human–animal interactions, Human–animal relationship, Naturalistic interactions, Positive animal welfare, Animal behaviour, Psychophysics

## Abstract

Positive human–animal interactions (HAIs) can be intrinsically rewarding and facilitate positive human–animal relationships. However, HAI paradigms vary across studies, and the influence of different interaction paradigms on the animal’s response has been overlooked. We compared the behavioural responses of pigs (*n* = 28) individually tested with two types of gentle tactile interactions with a familiar human: ‘free form (FF)’ where the pig could voluntarily approach and interact as they normally would, and ‘imposed contact (IC)’ where the human imposed tactile contact on the pig according to a standardised protocol. Pigs did not differ in their level of engagement with the human between the two types of interactions. However, they differed in their behaviour as they explored the pen more during the FF test, while they emitted more low-pitched vocalisations (grunts) during the IC test. These differences can likely be imputed to the IC test differing to the pigs’ habituation to human contact, which could have evoked greater attention to the human or triggered frustration due to violation of expectation. These findings highlight the influence of the predictability of the interaction or level of agency provided to the animal in HAI tests and relation to their previous experience of interacting.

## Introduction

Human–animal interactions (HAIs) can have a profound impact on animal welfare^1,2^, and this is reflected in the inclusion of positive HAIs in various animal welfare frameworks^3,4^. Human–animal interaction studies since the 1970s have predominantly focused on the impacts of negative HAIs^5^. Recent years have seen a paradigm shift in views of animal welfare, with a push for a greater emphasis on positive animal welfare^6,7^.

There is a growing body of literature supporting the potential benefits of positive HAIs. Positive HAIs can both be intrinsically rewarding and facilitate positive human–animal relationships (HARs), a relationship that is established when the animal forms expectations of its interactions with humans, and that can change based on subsequent interactions^8^. Provision of gentle tactile interactions can reduce animals’ fear of humans and increase approach behaviour, suggesting a positive perception of the interaction by the animal (pig^9,10^; cow^11,12^; dog^13^; sheep^14^). The incorporation of positive HAIs can also be used as an enrichment strategy in zoo and laboratory settings for its anxiolytic effects and ability to elicit positive behavioural changes (zoo^15,16^; laboratory^17,18^). Effects can also be seen extending beyond the interaction^8^. For example, positive human contacts can reduce incidences of abnormal behaviour such as tail biting in pigs^19^, increase grooming and reduce oral abnormal behaviours amongst chimpanzees^20^, and decrease heart rate in cows, indicating a relaxation effect post-stimulus^21^.

Nonetheless, it is still unclear what makes an interaction qualify as positive from the animal’s point of view^8^. Due to the inherently complex and multidisciplinary nature of HAIs, studies remain largely varied in their theoretical approaches, and methodologies are highly context-dependent^22,23^. Some studies have investigated animals’ responses to gentle contact on different body regions (cow^24,25^; pig^26^), whilst others have investigated the type of contact given by the human, such as comparing stroking and scratching in piglets^26^, or the frequency and duration of contact, such as tickling in rats^27^.

Interaction protocols also differ. Some studies use structured interactions, *i.e.* the interacting human follows a predetermined, standardised protocol (usually ‘imposed contact’) (dog^28^; pig^26,29,30^; cow^31^), whilst others employ unstructured (‘free form’) interactions, i.e. the interacting human may be given no specific instructions or is told to interact as they normally do (chimpanzee^20^; marmoset^32^, dog and robot^33^). In humans, neural and cognitive processes differ when interactants engage in truly interactive contexts compared to constrained and unnatural contexts that are commonly used in experimental research^34^. Therefore, an unstructured, free form interaction that allows for the breadth of responses from both interactants could help to truly understand the underpinnings of social interactions^34–36^. To our knowledge, only one study has compared structured and unstructured human–animal interactions^37^, where the structured interaction involved the child and dog moving through an agility arena, and the unstructured interaction involved the child and dog freely playing together (e.g. with a ball). They did not find significant differences in the behavioural indicators of positive affect shown by either the children or the dogs between these two types of interactions, and it is unknown whether these results translate to other types of human–animal interactions.

Pigs hold a unique place in our society as they are commonly kept as farmed^38^, laboratory^39^, or companion animals^40^. They are highly social with conspecifics and humans^41^, making them a suitable model species for the study of HAIs. Their increasing popularity across multiple contexts renders them susceptible to the impacts of HAIs on a large scale, and thus it is imperative to gain a better understanding of pig-human interactions in order to benefit their welfare^42^.

In this study, we compared two types of gentle tactile interactions: (1) ‘free form’ (FF), characterised by an unstructured interaction where the pig is allowed to voluntarily approach and interact, and the human is responsive to the pig’s solicitations, and (2) ‘imposed contact’ (IC), characterised by a structured interaction where the human imposes contact on the pig in a standardised manner and the human is irresponsive to the pig’s solicitations. The tests were conducted using a within-subject design over two consecutive days with a counterbalanced order.

We hypothesised that pigs would show signs of a more positive perception of HAIs if they are allowed to choose when and how to interact (FF) as opposed to when contact is imposed by the human (IC). The pig’s response was assessed based on indicators of engagement (approach behaviour and active contact by the pig) and positive affect (tail wagging). We also expected more resting behaviour and positive social interactions with pen mates following free form HAIs.

## Results

All results are presented as estimated mean ± standard error.

### Behaviours during the human–animal interaction test

#### Activity

**Fig. 1:**
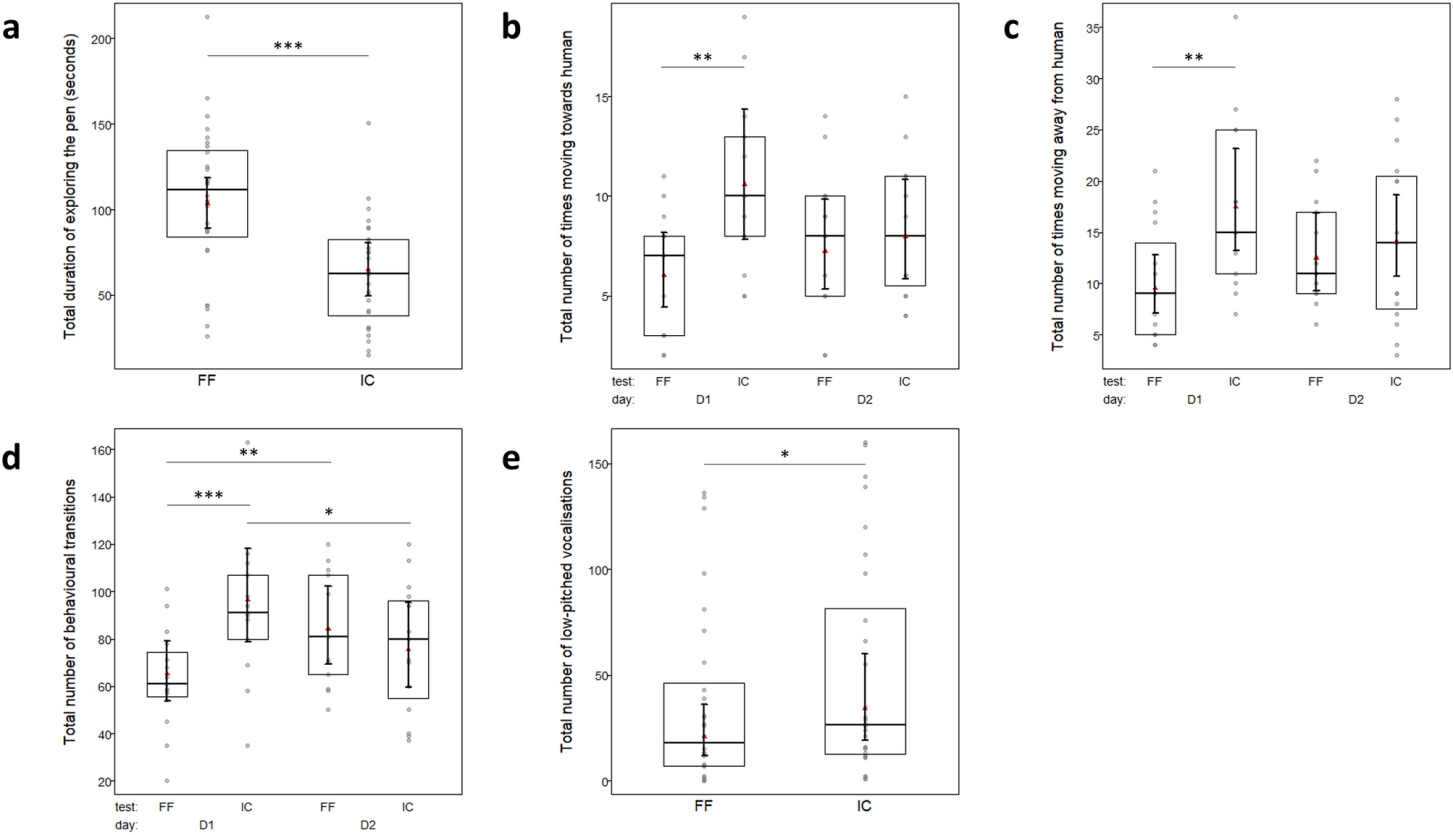
Behaviours of pigs (*n* = 28) during the test sessions for which test type or the interaction of test type × day were statistically significant based on post-hoc pairwise comparisons. (**a**) Total duration of exploring the pen; (**b**) Total number of times moving towards the human; (**c**) Total number of times moving away from the human; (**d**) Total number of behavioural transitions; (**e**) Total number of low-pitched vocalisations. Red triangle = estimated mean; horizontal line within box = median; lower and upper edge of box = 1st and 3rd quartile; error bars = 95% confidence intervals; grey dots = raw data points. *FF* free form, *IC* imposed contact, *D1* day 1, *D2* day 2. *p < .05; **p < .01; ***p < .001.

Pigs spent significantly more time exploring the pen during FF than IC (103.8 ± 7.3 s vs. 64.9 ± 7.4 s, F_1,21_ = 24.35, *p* < 0.001, Fig. 1A), but there was no significant effect of day (*p* = 0.395) or its interaction with test type (*p* = 0.416).

Although not statistically significant, pigs moving towards and moving away from the human showed a strong tendency to differ according to the interaction of test type × day (moving towards: z = − 1.95, *p* = 0.051; moving away: z = − 1.91, *p* = 0.057), where pigs moved towards and away from the human significantly less during FF than IC on day 1 (moving towards: z = − 2.32, *p* = 0.003, Fig. 1B; moving away: z = − 3.51, *p* = 0.003, Fig. 1C) but not on day 2 (moving towards: *p* = 0.949; moving away: *p* = 0.903).

No other active behaviours were significantly influenced by test type, day, or their interaction (Supplementary Table S1).

#### Behavioural transitions

The number of behavioural transitions differed significantly according to the interaction of test type × day (z = − 4.02, *p* < 0.001, Fig. 1D), where the number of transitions was significantly lower during FF compared to IC on day 1 (z = − 4.55, *p* < 0.001) but not on day 2 (*p* = 0.728), and significantly lower during FF on day 1 compared to FF on day 2 (z = − 3.32, *p* = 0.005), but significantly higher during IC on day 1 compared to IC on day 2 (z = 2.61, *p* = 0.044).

#### Latencies to approach and contact

The pigs’ latencies to approach and to make first contact with the human did not significantly differ according to the type of test, day, or their interaction (Supplementary Table S1).

#### Vocalisations

The total number of low-pitched vocalisations was significantly lower during FF than IC (20.6 ± 5.9 times vs. 34.0 ± 9.9 times, z = − 2.01, *p* = 0.045, Fig. 1E), with no significant effect of day or its interaction with test type. The probability of high-pitched vocalisations being emitted varied significantly between days (z = 3.766, *p* < 0.001), being significantly higher on day 1 than day 2 (probability: 1.0 ± 0.0 vs. 0.0 ± 0.0, *p* < 0.001), but with no significant effect of test type (*p* = 0.785) or its interaction with day (*p* = 0.732).

#### Tail posture and movement

Although not statistically significant, pigs tended to spend less time with their tail curled during FF than IC (274.0 ± 5.0 s vs. 280.0 ± 5.1 s, z = − 1.70, *p* = 0.095), and on day 1 than day 2 (274.0 ± 4.9 s vs. 281.0 ± 5.1 s; z = − 1.90, *p* = 0.060), but there was no significant effect of the test type × day interaction. No other tail posture or movement were significantly influenced by test type, day, or their interaction (Supplementary Table S1).

### Influence of the duration of contact by human

The duration of contact by the pig was significantly higher with longer duration of contact by the human (F_1,13_ = 66.94, *p* < 0.001), while pigs spent significantly more time immobile (F_1,45_ = 10.426, *p* = 0.002) and significantly more time with their tail curled (z = − 2.20, *p* = 0.034) with shorter duration of contact by the human. Although not statistically significant, pigs also tended to spend more time exploring the pen with shorter duration of contact by the human (F_1,49_ = 3.19, *p* = 0.080).

### Post-test behaviours

Agonistic behaviour between pen mates after returning from the test differed according to the test type × day interaction (F_1,26_ = 4.43, *p* = 0.045), but post-hoc pairwise comparisons were not statistically significant. Pigs tended to perform less behavioural transitions after the FF test than after the IC test (128 ± 20.1 vs. 153 ± 20.0, F_1,15_ = 4.06, *p* = 0.062), but there was no significant effect of day or its interaction with test type. No other behaviours observed post-test were significantly influenced by test type, day, or their interaction (Supplementary Table S2).

### Assessment of the human–animal relationship

All pigs reduced their latency to approach and their avoidance distance from the interacting human after the end of the testing period compared to before habituation. The median latency to approach was 300 s (range: 25–300 s) before habituation and 7 s (range: 2–80 s) after the end of the testing period. The respective median avoidance distances were 0.5 m (range: 0–3 m) and 0 m (range: 0–1 m). Therefore, these results confirmed that all pigs had built a positive human–animal relationship with the familiar human.

## Discussion

Contrary to our predictions, pigs showed neither higher engagement nor greater signs of positive affect in the FF test compared to the IC test, based on the amount of contact initiated by the pig, approach behaviour, and tail wagging. However, there was a difference in their behaviour in that pigs explored the pen more during the FF tests and emitted more low-pitched vocalisations (grunts) during the IC tests. There was also a subtle influence of the test type × day interaction on the number of behavioural transitions that suggests an influence of test sequence and previous experience. Altogether, these differences indicate a difference in the pigs’ perception of these different types of interactions that is likely attributed to a violation of expectation formed by their previous experience of interacting with the human or due to the change in their level of agency over the interaction.

Pigs did not differ in their time spent actively making contact with the human, suggesting a similar level of engagement despite the different interaction approaches used in the tests. We designed the duration of contact given by the human in the IC test to result in a similar duration of contact as the FF treatment (based on previous unpublished data) in order to avoid confounding effects of interaction styles and duration of interactions. Despite controlling the human’s behaviour, the pigs were still free to initiate contacts with the human in the non-responsive periods of the IC sessions. The significant effect of the contact by human covariate on the duration of contact by pig, standing or sitting immobile, and curled tail suggests that the act of the human giving contact was associated with changes in the pigs’ behaviours, although the direction of influence (i.e. whether the pig influenced the human or human influenced the pig) cannot be ascertained based on these results. The average duration of contact by the pigs was approximately one third of the test session during the IC condition, similar to Tallet et al*.*^26^ using structured interactions in which this was considered to be a high level of interest in the human. For the FF condition, the average duration of contact by the pigs was approximately one quarter of the test session, similar to Villain et al*.*^43^ using unstructured interactions where the human also interacted in the same way as they did during habituation. It is worth noting that direct comparisons between studies are difficult to make due to differences in the parameters used to indicate interest in the human, such as the type of behaviour or distance from the human^44–46^.

Our results do not suggest that pigs have a clear preference for one type of interaction over another, as they did not differ in terms of our indicators of engagement (i.e. contact by pig, moving towards human) and positive affect (i.e*.* tail wagging) in the way we predicted. This is consistent with the findings of a previous study that did not find significant differences in positive affect when comparing structured and unstructured dog–human interactions^37^. Pigs did not differ in their latency to move towards the human and make first contact, unlike previous studies where pigs showed more approach behaviour when the human was squatting and not approaching versus standing and approaching^44,47^. This could be due to the differences in habituation procedures, where our pigs were exposed to the human standing and approaching at several points during the habituation period to avoid fearful reactions whereas other studies did not^44,47^. We did not observe any differences between the two test conditions for tail wagging, hanging tail, or tucked tail, despite their links to affective states in previous studies^48–51^. Although there tended to be a difference in duration of curled tail between test conditions and between days, we deemed these 2.2% and 2.5% differences respectively to be not biologically significant. As the pigs’ tails were curled for the majority of the time in both test conditions, this is consistent with curled tail being considered a default, neutral posture^48^. Hanging and tucked tail were rare postures in our study. The lack of differences in tail posture and movement could be due to the fact that both conditions in our study were designed to be positive gentle interactions, so perhaps either the differences were too subtle to observe or the conditions were not dissimilar enough to produce an observable difference compared to other studies where they have compared situations of opposite valence (negative vs. positive)^49^ or investigated tail wagging in specific contexts (play^50^; exploration^51^).

We observed differences in other active behaviours that may reveal more information about the pigs’ perception of the tests and their underlying motivations. The higher duration of exploration during FF compared to IC interactions suggests that the pigs preferred to spend more time exploring the pen when they were free to choose when to interact with the human. The underlying motivation for this higher duration of exploration in the FF test, as a non-human-directed behaviour, remains ambiguous given that the environment was familiar to the pigs in both conditions. Perhaps the pigs increased their attention towards the human during the IC test because the imposed contact by the human was novel to the pigs. Thus, the lower duration of exploring the pen in the IC test compared to the FF tests could be explained by the increase in pigs’ behaviours relating to attention towards the human such as moving towards and moving away from the human.

The effect of test type on behaviours related to attention and responsiveness to the human (i.e. moving towards and moving away) was more pronounced on day 1, which suggests an influence of previous experience. It is plausible that the pigs who underwent IC on day 1 were paying more attention to the human even in their subsequent FF test on day 2 due to their experience from the previous day, thus attenuating the difference between conditions on day 2. Likewise, pigs who started with the IC test on day 1 performed a higher number of behavioural transitions during the IC test on that day as well as during the FF test on the subsequent day compared to the group that started with FF on day 1, suggesting an influence of test sequence and previous experience. These results combined with the production of more low-pitched vocalisations during the IC tests suggests that this test condition was perhaps mildly stress-inducing^52^ or frustrating^26,53^. This is consistent with similar studies where an irresponsive, immobile familiar human seemed to induce frustration in pigs as the test did not match what they previously experienced with the human^26,29^, or when pigs were not rewarded for a behaviour they were previously conditioned for^54,55^, i.e*.* violation of expectation (cetacean^56^; dog^57^) or affecting their level of perceived agency^58^. However, as low-pitched vocalisations can occur in both positive and negative situations^59^, we cannot conclude on the valence of our test situation based on our results. Other studies have suggested that the increased number of vocalisations could have a communicative function, as the vocalisations could be contact calls to conspecifics^60^, or attempts to seek information from a familiar human when their characteristics have been altered compared to the pigs’ previous experience^61^.

An alternative explanation for the increased number of moving towards and moving away during the IC test is that this could resemble the approach-avoidance conflict invoked during human interaction tests such as the human approach test and withdrawal response test described in Murphy et al*.*^62^. The IC test as we designed it is essentially a combination of these two tests, where during the no contact phase the human remains immobile like in the human approach test, and during the approaching phase the human walks towards the pig like in the withdrawal response test. Although the human is familiar in our tests, the change in behaviour during the IC test could be an aspect of novelty that induces curiosity during the no contact phase whilst invoking avoidance during the approaching phase. Nevertheless, the pigs did not show escape attempts, which suggests the IC test did not induce major distress in the pigs.

As a limitation to our study, the experimenter mainly responsible for data collection and analyses (S.T.) was involved in the study design (*i.e.* knew the hypotheses) and acted as the interacting human during the tests. Blinding this person was not possible, which could have biased the human interactions and, therefore, the results. However, as there were no significant effects of test type, day, test type × day interaction, and batch on the duration of contact by the human (Supplementary Table S1), it is unlikely that the behaviour of the human differed, suggesting that the human behaved consistently. Additional limitations of our study include the small sample size, which could have been insufficient to detect potential subtle differences given the inter-individual variability on the behaviours recorded, and the involvement of only one interacting human for all pigs, which limits the generalisation of the results. Therefore, the results obtained in the current study encourage further investigations, using a larger sample size and several interacting humans.

## Conclusion

Pigs showed subtle differences in their response to gentle tactile interactions with a familiar human depending on whether they could choose when and how to interact versus when contact was imposed on them. Although the pigs did not appear to show a clear preference for either type of interaction based on indicators of engagement and affective states, their behavioural responses suggest that changes in a familiar human’s behaviour made them more attentive towards the human and possibly induced frustration due to expectancy violation or reduced agency. These findings highlight the influence of potential expectations formed by previous experiences on the animal’s response and how the design of interaction protocols can impact study outcomes.

## Methods

This study was approved by the Ethics and Animal Welfare Committee of the University of Veterinary Medicine Vienna (project number ETK-182/12/2020) and all methods were carried out in accordance with Good Scientific Practice guidelines and national legislation. All methods are reported in accordance with ARRIVE guidelines. This experiment does not fall under the requirement for human ethics (or human accordance) approval. We only had one person acting as the interacting person with the pigs, and this was one of the authors (Suzanne Truong), who provided informed consent.

### Animals, housing and feeding

The present study was conducted at the research farm of the University of Veterinary Medicine Vienna (VetFarm ‘Medau’) with a total of 30 pigs (Large White x Pietrain breed) over two batches of 15 pigs each. Before weaning, sows and their piglets were housed in BeFree farrowing pens (Schauer Agrotronic GmbH, Prambachkirchen, Austria) of 2.22 m × 2.86 m (sow movement area of 4.2 m^2^) with a plastic slatted floor and a concrete lying area. Pigs’ tails were kept intact. At weaning, i.e. approximately 28 days of age, 15 female pigs from three litters (i.e. 5 females per litter) were recruited based on being visually healthy and avoiding large variations in weight. They were housed in groups of three pigs (i.e. one pig from each litter, so an equal representation of litters in each pen) in weaner pens (2.45 m × 3.20 m) consisting of grey plastic walls, concrete partly-slatted flooring (slatted area = 2.45 m × 1.23 m; 40% of pen area) equipped with a multi-place feeder (four places), one drinker bowl, and a shelter area (2.45 m × 1.00 m) with a roof 0.94 m above the ground. Pen enrichment consisted of straw and sawdust in the non-slatted area of the pen, a braided jute rope (1 m long) hung from the pen door and an orange dog toy ball (Airflow ball, Dog Crest, 7.6 cm diameter). Spot cleaning (removal of faeces in the non-slatted area) of the pens occurred when needed, and deep cleaning (removal of faeces in all areas of the pen) and refreshment of straw and sawdust was done twice weekly. Room temperature was automatically regulated around 22 °C (mean ± standard error: 21.8 ± 0.08 °C (range: 16–30.2 °C) in batch 1; 22.0 ± 0.11 °C (range: 14.8–29.7 °C) in batch 2) through a temperature control system (CB2012, Strijbos Climatech, de Wit AgroHandel GmbH; Austria), and thermal comfort of the pigs was ensured by floor heating. The room windows provided natural lighting to all the pens and artificial lights were turned on daily from 08:00 until 15:00. The experimenters always knocked on the door before entering the room to avoid startling the pigs. Throughout the experimental period, pigs were fed a standard weaner diet (17.5% crude protein, 7% crude fat; Garant-Tiernahrung GmbH, Austria) topped with a supplement (Biotronic Top3 and ProbioBac; Biomin®, Austria) provided ad libitum.

### Experimental design

Two female experimenters conducted the experiment. The first experimenter (S.T.: 159 cm, wearing black overalls and black boots) was the ‘interacting human’. The second experimenter (O.S.: 173 cm, wearing red overalls and black boots) was the ‘handler’, who moved the pigs between their home pen and the test pen. Farm staff were also female and wore either red, dark blue or dark green overalls. Pigs were marked by the handler on their back with an animal marker spray to facilitate individual identification, and re-marking always occurred at the end of the day when necessary.

### Assessment of the human–animal relationship

Human voluntary approach and human avoidance tests were conducted once before the habituation period and once after the test period in order to verify the establishment of a positive human–animal relationship, and check for individual differences in the human–animal relationship. Both tests were conducted by the interacting human, as the study focus was on positive human–animal relationship with a specific human and not on the generalisation of that relationship to other humans.

For the human voluntary approach, the interacting human entered the home pen, walked to the opposite corner of the pen gate and stood still, waiting until the pigs touched her (i.e. snout in contact with boot or overall). The latency for each pig to touch the interacting human was recorded. The test ended when all pigs touched the interacting human or after 5 min.

For the human avoidance test, one pig per group was tested at a time. The interacting human chose a focal pig at random, moved to a starting location within the home pen around 3 m away from them, and moved towards them continuously in steps of 0.5 m at a speed of one step per second until the pig moved to avoid the experimenter (defined as the pig moving both of its front feet). The distance to which the interacting human could approach the pig before it moved was estimated in increments of 0.5 m. If the pig did not move, the interacting human attempted to touch it on the forehead and, if successful, the distance recorded was 0 (if not successful, the distance recorded was 0.5 m). The test was attempted up to three times in case the pig moved prior to the experimenter placing herself at 3 m to start the test. A 5 min break was observed between rounds of human avoidance test in a pen, and between the human approach test and the human avoidance test.

### Habituation

**Fig. 2:**
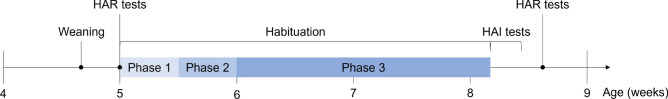
Experimental timeline. HAR: human–animal relationship. HAR tests: human approach and avoidance tests. Habituation to the interacting human and testing environment consisted of 2 sessions per day (excluding weekends) in three phases: Phase 1—group habituation in the home pen; Phase 2—group habituation in the test pen; Phase 3—individual habituation in the test pen. HAI: human–animal interaction.

Starting from 3 days post-weaning (at 5 weeks of age), all pigs were gradually habituated to human presence, human contact, and to the test pen over 3 weeks in a stepwise process (Fig. 2). The interaction procedure during all phases of habituation (unless specified otherwise) was as follows: the experimenter entered the pen, kneeled in the corner opposite to the door and attracted their attention by talking softly to them, encouraging the pigs to approach. If the pigs initiated contact with her, she presented her hand to them and then gently stroked and/or scratched them. These tactile contacts were first applied on the head area (snout, head front and sides, neck) and slowly moved towards the rear of the body, including belly-rubbing if the pigs rolled on their back and exposed their belly^63^. Touching the ears of the pigs was avoided as it might be perceived as unpleasant (personal observations). Sudden movements and speaking in a loud voice were avoided. However, if a pig started to bite, the first reaction was to withdraw from the pig, and if it insisted it was gently tapped on their snout to discourage this behaviour.

In the first phase of the habituation, pigs were exposed to human presence and contacts in their home pen across two daily sessions of 5 min each over 2 days. Both experimenters were involved on the first day, and only the interacting human was involved from the second day onwards. This was to avoid fearful reactions to the handler while allowing more time for the pigs to develop a positive relationship with the interacting human in the tests. Each experimenter entered the home pens separately and kneeled in the opposite corner of the pen.

In the second phase of the habituation, each group was guided by the handler to the test pen across two daily sessions of 5 min over 2 days. The handler exited the room after bringing the pigs to the test pen, and only returned to the room when the 5 min had elapsed. The interacting human was already in the test pen, kneeling in the corner opposite to the door. On the second day, the interacting human stood up and walked towards the pigs at the speed of approximately one step per second twice during the session in order to habituate them to being approached and avoid fear reactions during the imposed contact test sessions. Upon approaching the pigs, the interacting human kneeled and allowed the pigs to voluntarily approach and interact as per the interaction procedure for habituation.

In the last phase of habituation, each pig was moved individually to the test pen, where it stayed for a gradually increasing amount of time (from 1 to 5 min) across days. Sessions in this last phase were conducted twice daily over 5 days, then once daily over the last 6 days of habituation. The duration was increased only when the pig did not show any signs of distress. The following behaviours were considered signs of distress: three acts of defecation or urination, five high-pitched vocalisations within 1 min, three attempts to escape, or a combination of any two of these behaviours. At any phase of the habituation, the session was ended immediately if a pig showed distress reactions and it (or the group of pigs) was returned to the home pen. In case distress reactions happened during the first sessions of the last phase (i.e. individual habituation), pigs underwent one session in their group and one individual session on the next days until they no longer showed signs of distress.

On the last two sessions, the interacting human once again habituated the pig to being approached. Individual preferences for the type and region of tactile contact were recorded during the last phase of habituation, to facilitate the delivery of contact that was preferred by each individual pig during the FF test session. Preferences for tactile contact was assessed by the interacting human based on the lack of withdrawal response and maintained contact by the pig. Additionally, during the last 4 days of habituation, a microphone was installed in the middle of the pen (suspended 1 m above the ground for the recording of pig vocalisations) to familiarise the pigs with it.

### Human–animal interaction tests

After three weeks of habituation, all pigs were subjected to two 5-min tests across two consecutive days using a within-subject, counterbalanced design. Each pig underwent each of the two different types of HAIs (Table 1), with half the pigs starting with the free form contact (FF) condition and the other half starting with the imposed contact (IC) condition on the first day, then undergoing the alternate test type on the next day. Pig behaviour was video recorded during the test sessions and in their home pen for 30 min post-test. The test order of pigs was pseudo-randomised within each pen group and within a day to avoid order effects, ensuring that there was at least 30 min between two pigs of the same pen to avoid disturbance of the post-test recording. This test order was the same for both tests to avoid an effect of time. To avoid potential rebound effects after a prolonged period without human contact over the weekend, a session of habituation was conducted on the Monday and the tests were conducted on the following days. Sessions took place in the morning between 8:30 and 11:30 h when the pigs were most active.

**Table 1:** Description of the types of interaction provided by the interacting human during the 5-min interaction test session (see Videos in the Supplementary Material).

Test condition	Description
Free form contact (FF)	The interacting human remains kneeling in the corner opposite to the pen gate for the duration of the session. The pig is allowed to approach and interact on a voluntary basis. The human encourages the pig to approach using a soft talking voice, and small hand and arm gestures such as tapping of the fingers. If the pig is within arm’s reach, the interacting human provides a variety of gentle tactile contact including stroking, scratching, and rubbing as she had done during the last phase of habituation, responding to the pig’s solicitation cues
Imposed contact (IC)	The interacting human follows a standardised protocol from a pre-recorded audio playback playing through a wireless earbud, indicating the timing of a contact sequence repeated 10 times: Approaching (8 s): If the pig is not within arm’s reach, the human stands up and walks towards the pig at a speed of one step per second and kneels down within 8 s. If the pig is already within arm’s reach, the human remains kneeling with arms folded and placing her gaze on the far wall to avoid eye contact, ignoring the pig until the last 2 s when she orientates her body towards the pig Imposing contact (15 s): The interacting human presents her hand to the pig’s snout in the first second, followed by stroking from the neck to the lower back at a rate of one stroke every 2 s Withdrawing from the pig (7 s): The interacting human stands up and walks away from the pig to the corner of the pen opposite to the door, kneels, folds her arms, places her gaze on the far wall and ignores the pig, waiting for the next contact bout The human verbalises each of her actions during the sequence: “Standing”, “Walking”, “Kneeling”, and says “Good girl” when applying each stroke on the pig

The testing procedure was as follows: the handler moved the pig from the home pen to the test pen and exited the room. The interacting human then entered the room, said “hi piggy” upon arriving at the test pen gate, then entered the pen and locked the pen gate. The interacting human proceeded to walk from the pen gate to the corner opposite the door, the first step initiating the 5-min session. She then kneeled in the corner and followed the procedure outlined in Table 1 according to the type of interaction. Once 5 min elapsed, the handler re-entered the room and moved the pig back to its home pen, where its behaviour was recorded for a further 30 min to observe post-test behaviours.

The IC sequence (Table 1) resulted in a maximum of ten interactions over the 5-min test session (15 s × 10 min = 150 s or 50% of test time), which was designed to be similar to the amount of contact time obtained in a previous trial where FF contact was employed (unpublished data). During the IC tests, if the pig refused the interaction, i.e., by moving two steps away from the human during the approach, then the interacting human immediately returned to the initial location, kneeled, and waited until the next bout to engage in a new contact bout.

### Behavioural analyses

The test pen and the home pens were continuously video recorded (camera Hikvision DS-2CD5046GO-AP; Hangzhou Hikvision Electronics Co., Ltd.; Hangzhou, China) from 8:00 to 17:00 h, which allowed behavioural analysis during test sessions and post-test with the BORIS software^64^ (version 7.10.2) using continuous focal sampling^65^. It was not possible to blind the observer to the type of interaction, however inter- and intra-observer reliability were assessed and deemed acceptable.

### Behaviours during test sessions

The behaviours of the pigs during the 5-min tests were recorded using an ethogram (Table 2), with a particular focus on behaviours toward the human, indicators of engagement (i.e. contact by the pig, moving towards), and indicators of affective state (i.e. tail posture and movement, vocalisations, distress-related behaviours). Latencies to first approach and first contact by the pig were also recorded, and the number of behavioural transitions was calculated as the sum of changes between behavioural states. Additionally, we recorded the duration of contact by the human to check the consistency of the human’s behaviour as the tests were designed so that the human would deliver different types of contact but overall similar total durations of contact in both test conditions.

**Table 2:** Ethogram for behavioural observation of the 5-min test sessions. Behaviours within the same category are mutually exclusive unless stated otherwise. Behaviours were coded as state events unless stated otherwise. Definitions adapted from other studies are cited.

Category	Behaviour	Definition
Activity	Contact by pig	Pig makes active contact with human. Includes sniffing, nosing, oral manipulation, rubbing, pawing
	Body contact^26^^,a^	Pig makes body contact with human. Includes climbing on human, standing with body against human, and lying or sitting on human
	Contact by human^b^	Pig receives active contact (stroking, scratching, rubbing) by the human
	Following	Pig takes more than two steps in the same direction as the moving human
	Moving towards	Pig takes more than two steps in a direction that decreases the distance between pig and human
	Moving away	Pig takes more than two steps in a direction that increases the distance between pig and human
	Exploring the pen^c^	Pig’s snout touches pen floor or wall for more than 1 s
	Lying down^d^	Pig’s thorax touches the ground
	Immobile	Pig is either sitting or standing for more than 2 s and less than 30 s, shows no specific movement
	Inactive (Sara Hintze, unpublished)	Pig is either standing or lying, shows no specific movement for more than 30 s. Pig remains inactive if it engages in exploration maximum twice or for less than 5s, takes less than five steps away, stretches or changes posture
	Escape behaviour^c^	Pig nudges/pushes against pen door using snout or front legs; climbs door, wall or into back corner
	Escape attempt^e^	Pig jumps against the door or wall
	Elimination^e^	Pig excretes faeces or urine
	Not visible	Pig’s behaviour cannot be determined due to snout being obstructed from view
Tail posture and movement^66^	Hanging tail	Tail is pointing downward
	Curled tail	Tail coiled up in a curl on top of the body
	Tucked tail	Tail is kept vertically down and close to the body whereby the tip of the tail is held between the legs
	Wagging	Tail swinging in any direction, but mostly from side to side
	Not visible	Pig’s tail posture or movement cannot be determined due to tail being obstructed from view
Vocalisations^26^	Low-pitched vocalisation	Grunts
	High-pitched vocalisation	Grunt-squeals, squeals, and screams

^a^‘Body contact’ did not exclude ‘Contact by pig’, ‘Contact by human’, ‘Immobile’, ‘Inactive’, ‘Other’, and ‘Not visible’.

^b^‘Contact by human’ did not exclude any behaviours.

^c^An interruption of > 3 s was considered a new bout.

^d^‘Lying down’ did not exclude ‘Contact by pig’, ‘Contact by human’, ‘Exploring’, ‘Inactive’, ‘Other’, and ‘Not visible’.

^e^Scored as point event due to short duration.

### Audio recording during test sessions

Pig vocalisations during the test sessions were recorded with a microphone (Sennheiser MKE600; Sennheiser electronic GmbH & Co. KG, Wedemark, Germany) linked to a recorder (Zoom H4N; ZOOM Corporation, Tokyo, Japan). In order to match the time in the audio recording to the time in the video recording, the interacting human tapped the microphone after each session. Vocalisations were coded as point events in the BORIS software^64^ (version 7.10.2) according to two categories: low-pitched (grunts) and high-pitched (grunt-squeals, squeals and screams).

### Behaviours after the test sessions (post-test)

At the beginning of each testing day, the roof of the shelter area in each home pen was removed to allow recording of the pigs’ behaviour post-test. The post-test behaviours (Table 3) in the home pen were observed for 30 min using continuous focal sampling^65^, with a special focus on resting (i.e. inactive, lying down) and social behaviours (i.e. social play, snout to snout contacts) of the previously tested pig.

**Table 3:** Ethogram used for the behavioural observations post-test interaction during 30 min in the home pen. Behaviours were coded as state events unless stated otherwise. Interruption of more than 2 s was considered a new bout. Definitions adapted from other studies are cited.

Category	Behaviour	Definition
Activity	Eat or drink	Focal pig has her head in the trough or in the drinker
	Eliminates^a^	Focal pig excretes faeces or urine
	Explores	Focal pig’s snout touches pen fitting
	Immobile	Focal pig is either sitting or standing for more than 2 s and less than 30 s, and shows no specific movement
	Inactive (Sara Hintze, unpublished)	Focal pig is either standing, sitting or lying, show no specific movement for more than 30 s. Focal pig remains inactive if it engages in exploration maximum twice or for less than 5 s, takes less than five steps away, stretches or changes posture
	Lying down	Focal pig’s thorax touches the ground
	Locomotor play	Focal pig produces erratic movements with no clear purpose, sudden reversal of behaviours. Includes run, spring, pivot. Performed alone
	Object play	Focal pig nudges, chews, pulls or shakes enrichment (ball or rope)
	Social play^67^	Focal pig is performing playful behaviour with, or towards, another pig. Includes scampering, running, pivoting, head tossing, flopping or hopping
	Agonistic behaviour	Focal pig forcefully fights with another pig. Includes bites, head knocks and chasing. Usually not performed within a play bout
	Nose to nose	Focal pig is actively touching another pig’s snout with its snout (initiating the interaction)
	Nose to body^67^	Focal pig is actively touching another pig’s body part with its snout (initiating the interaction)
	Receives	Focal pig receives interaction from another pig: agonistic, nose-to-nose or playful
	Not visible	Focal pig is not clearly visible (*i.e.* typically snout is hidden) or its behaviour cannot be determined

^a^Scored as point event due to short duration.

### Statistical analyses

Statistical analyses were performed using the R statistical software^68^ (version 4.3.3). We used (generalised) linear mixed models ((G)LMMs) to analyse all test and post-test behaviours using separate models for each behavioural variable. For LMMs and GLMMs based on the Poisson and binomial distributions, we used the ‘lme4’ package^69^ (version 1.1.35.1). For GLMMs based on the negative binomial and Gamma distributions, we used the ‘glmmTMB’ package^70^ (version 1.1.8). A global model was created including test type, day, and their interaction as fixed effects, as we expected the effect of test type on behaviour to change depending on the order of presentation of the tests. We included batch as an additional fixed effect to control for its potential effect. Individual, sow, pen, and group were included as random intercept effects. We included random slopes for day and test type within sow, day and test type within group, and test type × day interaction and batch within pen. For test behaviours except for latencies, we also included the duration of contact by the human as a covariate in order to account for the intrinsic variability between sessions due to the amount of contact given by the human.

Prior to fitting the models, we inspected all quantitative predictors and the response for whether their distributions were roughly normal. The variable latency to first ‘lying down’ for post-test behaviours was log-transformed to improve its distribution. The variables latency to first ‘moving towards’ and latency to first ‘contact by pig’ were transformed according to (y + 0.001) where y is the response variable to avoid values of zero as some pigs were already in contact with the human when the session started.

After fitting the models, we checked whether the assumptions of normally distributed and homogeneous residuals were fulfilled by visual inspection of a QQ-plot^71^ of residuals and residuals plotted against fitted values^72^. The plots indicated no deviations from these assumptions. We determined Variance Inflation Factors using the function vif of the ‘car’ package^73^ (version 3.1.2). Collinearity, determined for a model lacking the interaction, did not appear to be an issue (maximum Variance Inflation Factor: 1.04)^72^. Model stability was assessed by dropping the individuals one at a time from the data and comparing the estimates derived for models fitted to these subsets with those obtained for the full data set using a function provided by Roger Mundry. Models for the post-interaction variables and for the normally distributed test variables showed good stability. However, there were model convergence warnings for the models of all non-normally distributed test variables, and these results should be interpreted with caution. We used a parametric bootstrap (function ‘bootMer’ of the ‘lme4’ package^69^ (version 1.1.35.1); N = 1000 bootstraps) to obtain confidence intervals of model estimates and fitted values. We used the ‘emmeans’ package^74^ (version 1.8.9) to obtain the estimated means, standard errors and confidence limits of each level of the factors, as well as conduct multiple pairwise comparisons with Tukey adjustment.

As an overall test of the effects of test type, day, and their interaction, we conducted a full-null model comparison^75^, aiming at avoiding cryptic multiple testing, whereby the null model lacked the test type and day effects but kept the random effect of individual. This comparison was based on a likelihood ratio test^76^. We tested the effect of individual fixed effects by means of the Satterthwaite approximation^77^ using the function ‘lmer’ of the ‘lmerTest’ package^78^ (version 3.1.3) and a model fitted with restricted maximum likelihood. Due to the exploratory nature of this study, adjustments were not made for multiple testing and P values are suggestive of associations considering increased Type I error. The sample for each model encompassed 56 behavioural values, taken from 28 individuals out of two batches on 2 days. Two individuals (both from batch 2) were excluded from the analysis of test behaviours because their test sessions were terminated before 300s due to distress reactions. One individual (batch 2) was excluded from the analysis of latency to first contact by pig as it was the only pig who did not make any active contact with the human during both tests. An effect was considered significant if the P value was below 0.05, and a statistical trend if greater than 0.05 and less than or equal to 0.1. All results are presented as estimated mean ± standard error.

#### Test behaviours

All activity behaviours coded as state events were modelled using LMMs based on the Gaussian distribution unless stated otherwise. The behaviours ‘moving towards and ‘moving away’ were analysed for their total number instead of duration as the number reflects the decision of the animal to approach or avoid the human. The number of ‘moving towards was modelled using a GLMM based on the Poisson distribution with a log link function. The number of ‘moving away’, ‘transitions’, and ‘low-pitched vocalisations’ were modelled using a GLMM based on the negative binomial distribution with a log link function due to overdispersion. Due to the data being uncensored (i.e. all pigs approached and made contact with the human within the sessions), latencies were modelled with a GLMM based on the Gamma distribution with a log link function. The data for ‘escape behaviour, ‘body contact’, ‘high-pitched vocalisations’, ‘hanging tail’, and ‘wagging’ were dichotomised (occurrence: 1 = yes, 0 = no) and analysed using a GLMM based on the binomial distribution with a logit link function due to approximately half or more of the sessions with zero occurrences. The duration of ‘curled tail’ was modelled based on the Gamma distribution with a log link function due to data being left-skewed. For ‘escape behaviour’, number of ‘moving away’, latency to first ‘contact by pig’, latency to first ‘moving towards, ‘high-pitched vocalisations’, ‘hanging tail’, duration of ‘curled tail’, and ‘wagging’, a reduced model lacking random slopes and/or the ‘contact by human’ covariate was used due to model convergence issues with the full model. For some models, the ‘contact by human’ covariate was also z-transformed to aid model convergence. ‘Escape attempt’, ‘inactive’, ‘lying down’, and ‘tucked tail’ were excluded from the analyses as they were rare behaviours, occurring in less than five pigs. ‘Following’ and ‘moving towards’ were merged as they both described the pig moving in the direction of the human.

#### Post-test behaviours

All post-test activity behaviours coded as state events were modelled using LMMs based on the Gaussian distribution. Latencies to the first occurrence were calculated for targeted behaviours (inactive, lying down, social play, locomotor play and nose to nose) for each pig. When the behaviour did not occur during the observation time, latency was set to 1800s and censored. Data were then analysed with the non-parametric Kaplan–Meier survival analysis (Survfit), and the Cox regression analysis, using the packages ‘survival’^79^ (version 3.4.0) and ‘survminer’^80^ (version 0.4.9). The Kaplan–Meier survival curves and risk tables were produced only for the effect of test type, while the Cox regression analysis covered the effects of test type, day, and batch.

## Supplementary Information

Supplementary Video 1.

Supplementary Video 2.

Supplementary Information 1.

## Data Availability

Test data are provided in Supplementary Tables S3–S6, and post-test data are provided in Supplementary Tables S7–S9.

## References

[1] Waiblinger, S. et al. Assessing the human–animal relationship in farmed species: A critical review. *Appl. Anim. Behav. Sci. ***101**, 185–242. 10.1016/j.applanim.2006.02.001 (2006).

[2] Hemsworth, P. H. & Coleman, G. J. Human–animal interactions and animal productivity and welfare. in *Human-Livestock Interactions: The Stockperson and the Productivity and Welfare of Intensively Farmed Animals* 47–83. (2011).

[3] Mellor, D. J. et al. The 2020 five domains model: including human–animal interactions in assessments of animal welfare. *Animals ***10**, 1870. 10.3390/ani10101870 (2020). 33066335 10.3390/ani10101870 PMC7602120

[4] Leconstant, C. & Spitz, E. Integrative model of human–animal interactions: A one health-one welfare systemic approach to studying HAI. *Front. Vet. Sci. ***9**, 656833. 10.3389/fvets.2022.656833 (2022). 35968006 10.3389/fvets.2022.656833 PMC9372562

[5] Acharya, R. Y., Hemsworth, P. H., Coleman, G. J. & Kinder, J. E. The animal–human interface in farm animal production: Animal fear, stress, reproduction and welfare. *Animals ***12**, 487. 10.3390/ani12040487 (2022). 35203194 10.3390/ani12040487 PMC8868546

[6] Mellor, D. Enhancing animal welfare by creating opportunities for positive affective engagement. *New Zealand Vet. J. ***63**, 3–8. 10.1080/00480169.2014.926799 (2015). 24875268 10.1080/00480169.2014.926799

[7] Rault, J.-L., Hintze, S., Camerlink, I. & Yee, J. R. Positive welfare and the like: Distinct views and a proposed framework. *Front. Vet. Sci. ***7**, 370. 10.3389/fvets.2020.00370 (2020). 32714949 10.3389/fvets.2020.00370 PMC7343720

[8] Rault, J.-L., Waiblinger, S., Boivin, X. & Hemsworth, P. The power of a positive human–animal relationship for animal welfare. *Front. Vet. Sci. ***7**, 590867. 10.3389/fvets.2020.590867 (2020). 33240961 10.3389/fvets.2020.590867 PMC7680732

[9] Hemsworth, P. H. & Barnett, J. L. The effects of early contact with humans on the subsequent level of fear of humans in pigs. *Appl. Animal Behav. Sci. ***35**, 83–90. 10.1016/0168-1591(92)90018-7 (1992).

[10] Hayes, M. E., Hemsworth, L. M., Morrison, R. S., Tilbrook, A. J. & Hemsworth, P. H. Positive human contact and housing systems impact the responses of piglets to various stressors. *Animals ***11**, 1619. 10.3390/ani11061619 (2021). 34070802 10.3390/ani11061619 PMC8227335

[11] Hemsworth, P. H., Price, E. O. & Borgwardt, R. Behavioural responses of domestic pigs and cattle to humans and novel stimuli. *Appl. Animal Behav. Sci. ***50**, 43–56. 10.1016/0168-1591(96)01067-2 (1996).

[12] Lensink, B. J., Boivin, X., Pradel, P., Le Neindre, P. & Veissier, I. Reducing veal calves’ reactivity to people by providing additional human contact. *J. Animal Sci. ***78**, 1213. 10.2527/2000.7851213x (2000). 10.2527/2000.7851213x 10834574

[13] Conley, M. J., Fisher, A. D. & Hemsworth, P. H. Effects of human contact and toys on the fear responses to humans of shelter-housed dogs. *Appl. Animal Behav. Sci. ***156**, 62–69. 10.1016/j.applanim.2014.03.008 (2014).

[14] Sokołowski, J., Janicka, K., Zięba, G., Junkuszew, A. & Rozempolska-Rucińska, I. Effect of gentle physical contact on behavioural indicators in sheep. *Animal. ***17**, 100924. 10.1016/j.animal.2023.100924 (2023). 37611436 10.1016/j.animal.2023.100924

[15] Claxton, A. M. The potential of the human–animal relationship as an environmental enrichment for the welfare of zoo-housed animals. *Appl. Animal Behav. Sci. ***133**, 1–10. 10.1016/j.applanim.2011.03.002 (2011).

[16] Ward, S. J. & Melfi, V. The implications of husbandry training on zoo animal response rates. *Appl. Animal Behav. Sci. ***147**, 179–185. 10.1016/j.applanim.2013.05.008 (2013).

[17] Cloutier, S., Baker, C., Wahl, K., Panksepp, J. & Newberry, R. C. Playful handling as social enrichment for individually- and group-housed laboratory rats. *Appl. Animal Behav. Sci. ***143**, 85–95. 10.1016/j.applanim.2012.10.006 (2013).

[18] Baker, K. C. Survey of 2014 behavioral management programs for laboratory primates in the United States. *Am. J. Primatol. ***78**, 780–796. 10.1002/ajp.22543 (2016). 26971575 10.1002/ajp.22543 PMC4914436

[19] Büttner, K., Czycholl, I., Basler, H. & Krieter, J. Effects of an intensified human–animal interaction on tail biting in pigs during the rearing period. *J. Agric. Sci. ***156**, 1039–1046. 10.1017/S002185961800103X (2018).

[20] Baker, K. Benefits of positive human interaction for socially housed chimpanzees. *Anim. welf. ***13**, 239–245. 10.1017/S0962728600026981 (2004). 20505791 PMC2875797

[21] Lange, A., Bauer, L., Futschik, A., Waiblinger, S. & Lürzel, S. Talking to cows: Reactions to different auditory stimuli during gentle human–animal interactions. *Front. Psychol. ***11**, 579346. 10.3389/fpsyg.2020.579346 (2020). 33178082 10.3389/fpsyg.2020.579346 PMC7593841

[22] Hosey, G. & Melfi, V. Human–animal interactions, relationships and bonds: A review and analysis of the literature. *IJCP.*10.46867/ijcp.2014.27.01.01 (2014).

[23] Rodriguez, K. E., Herzog, H. & Gee, N. R. Variability in human–animal interaction research. *Front. Vet. Sci. ***7**, 619600. 10.3389/fvets.2020.619600 (2021). 33521092 10.3389/fvets.2020.619600 PMC7843787

[24] Schmied, C., Waiblinger, S., Scharl, T., Leisch, F. & Boivin, X. Stroking of different body regions by a human: Effects on behaviour and heart rate of dairy cows. *Appl. Animal Behav. Sci. ***109**, 25–38. 10.1016/j.applanim.2007.01.013 (2008).

[25] Lange, A. et al. Effects of different stroking styles on behaviour and cardiac parameters in heifers. *Animals ***10**, 426. 10.3390/ani10030426 (2020). 32143274 10.3390/ani10030426 PMC7143138

[26] Tallet, C. et al. Behavioural and physiological reactions of piglets to gentle tactile interactions vary according to their previous experience with humans. *Livestock Sci. ***167**, 331–341. 10.1016/j.livsci.2014.06.025 (2014).

[27] LaFollette, M. R., O’Haire, M. E., Cloutier, S., Blankenberger, W. B. & Gaskill, B. N. Rat tickling: A systematic review of applications, outcomes, and moderators. *PLoS ONE ***12**, e0175320. 10.1371/journal.pone.0175320 (2017). 28384364 10.1371/journal.pone.0175320 PMC5383284

[28] Rehn, T., Handlin, L., Uvnäs-Moberg, K. & Keeling, L. J. Dogs’ endocrine and behavioural responses at reunion are affected by how the human initiates contact. *Physiol. Behav. ***124**, 45–53. 10.1016/j.physbeh.2013.10.009 (2014). 24471179

[29] Terlouw, E. M. C. & Porcher, J. Repeated handling of pigs during rearing. I. Refusal of contact by the handler and reactivity to familiar and unfamiliar humans. *J. Animal Sci. ***83**, 1653–1663. 10.2527/2005.8371653x (2005). 10.2527/2005.8371653x 15956474

[30] Luna, D. et al. The effect of demonstrator social rank on the attentiveness and motivation of pigs to positively interact with their human caretakers. *Animals ***11**, 2140. 10.3390/ani11072140 (2021). 34359267 10.3390/ani11072140 PMC8300723

[31] Lange, A. et al. Effects of restraint on heifers during gentle human–animal interactions. *Appl. Animal Behav. Sci. ***243**, 105445. 10.1016/j.applanim.2021.105445 (2021).

[32] Manciocco, A., Chiarotti, F. & Vitale, A. Effects of positive interaction with caretakers on the behaviour of socially housed common marmosets (Callithrix jacchus). *Appl. Animal Behav. Sci. ***120**, 100–107. 10.1016/j.applanim.2009.05.007 (2009).

[33] Barber, O., Somogyi, E., McBride, A. E. & Proops, L. Children’s evaluations of a therapy dog and biomimetic robot: Influences of animistic beliefs and social interaction. *Int. J. Soc. Robot. ***13**, 1411–1425. 10.1007/s12369-020-00722-0 (2021).

[34] Redcay, E. & Schilbach, L. Using second-person neuroscience to elucidate the mechanisms of social interaction. *Nat. Rev. Neurosci. ***20**, 495–505. 10.1038/s41583-019-0179-4 (2019). 31138910 10.1038/s41583-019-0179-4 PMC6997943

[35] Dale, R., Yu, C., Nagai, Y., Coco, M. & Kopp, S. Embodied approaches to interpersonal coordination: Infants, adults, robots, and agents. (2013).

[36] Schilbach, L. et al. Toward a second-person neuroscience. *Behav. Brain Sci. ***36**, 393–414. 10.1017/S0140525X12000660 (2013). 23883742 10.1017/S0140525X12000660

[37] Pop, D., Rusu, A. S. & Miresan, V. The development of a canine para-agility program: Positive affects in children with autism and in therapy dogs. *BUASVMCN-ASB ***73**, 66–71. 10.15835/buasvmcn-asb:11812 (2016).

[38] FAOSTAT. Food and Agriculture Organization of the United Nations (FAO), Crops and livestock products. https://www.fao.org/faostat/en/#data/QCL (2024).

[39] Marchant-Forde, J. N. & Herskin, M. S. Pigs as laboratory animals. in *Advances in Pig Welfare* 445–475. 10.1016/B978-0-08-101012-9.00015-0 (Elsevier, 2018).

[40] Gerencsér, L., Pérez Fraga, P., Lovas, M., Újváry, D. & Andics, A. Comparing interspecific socio-communicative skills of socialized juvenile dogs and miniature pigs. *Anim. Cogn. ***22**, 917–929. 10.1007/s10071-019-01284-z (2019). 31256339 10.1007/s10071-019-01284-z PMC6834752

[41] Graves, H. B. Behavior and ecology of wild and feral swine (Sus Scrofa). *J. Animal Sci. ***58**, 482–492. 10.2527/jas1984.582482x (1984).

[42] Tallet, C., Brajon, S., Devillers, N. & Lensink, J. Pig–human interactions. in *Advances in Pig Welfare* 381–398. 10.1016/B978-0-08-101012-9.00008-3 (Elsevier, 2018).

[43] Villain, A. S., Lanthony, M., Guérin, C. & Tallet, C. Manipulable object and human contact: Preference and modulation of emotional states in weaned pigs. *Front. Vet. Sci. ***7**, 577433. 10.3389/fvets.2020.577433 (2020). 33330698 10.3389/fvets.2020.577433 PMC7728720

[44] Hemsworth, P. H., Gonyou, H. W. & Dziuk, P. J. Human communication with pigs: The behavioural response of pigs to specific human signals. *Appl. Animal Behav. Sci. ***15**, 45–54. 10.1016/0168-1591(86)90021-3 (1986).

[45] Day, J. E. L., Spoolder, H. A. M., Burfoot, A., Chamberlain, H. L. & Edwards, S. A. The separate and interactive effects of handling and environmental enrichment on the behaviour and welfare of growing pigs. *Appl. Animal Behav. Sci. ***75**, 177–192. 10.1016/S0168-1591(01)00199-X (2002).

[46] Lürzel, S., Bückendorf, L., Waiblinger, S. & Rault, J.-L. Salivary oxytocin in pigs, cattle, and goats during positive human–animal interactions. *Psychoneuroendocrinology ***115**, 104636. 10.1016/j.psyneuen.2020.104636 (2020). 32160578 10.1016/j.psyneuen.2020.104636

[47] Miura, A., Tanida, H., Tanaka, T. & Yoshimoto, T. The influence of human posture and movement on the approach and escape behaviour of weanling pigs. *Appl. Animal Behav. Sci. ***49**, 247–256. 10.1016/0168-1591(95)00658-3 (1996).

[48] Reimert, I., Bolhuis, J. E., Kemp, B. & Rodenburg, T. B. Indicators of positive and negative emotions and emotional contagion in pigs. *Physiol. Behav. ***109**, 42–50. 10.1016/j.physbeh.2012.11.002 (2013). 23159725 10.1016/j.physbeh.2012.11.002

[49] Reimert, I., Fong, S., Rodenburg, T. B. & Bolhuis, J. E. Emotional states and emotional contagion in pigs after exposure to a positive and negative treatment. *Appl. Animal Behav. Sci. ***193**, 37–42. 10.1016/j.applanim.2017.03.009 (2017).

[50] Rius, M. M. et al. Tail and ear movements as possible indicators of emotions in pigs. *Appl. Animal Behav. Sci. ***205**, 14–18. 10.1016/j.applanim.2018.05.012 (2018).

[51] Ocepek, M., Newberry, R. C. & Andersen, I. L. Which types of rooting material give weaner pigs most pleasure?. *Appl. Animal Behav. Sci. ***231**, 105070. 10.1016/j.applanim.2020.105070 (2020).

[52] Jensen, K. H. et al. Intermittent stress in pigs: Effects on behavior, pituitary—Adrenocortical axis, growth, and gastric ulceration. *Physiol. Behav. ***59**, 741–748. 10.1016/0031-9384(95)02159-0 (1996). 8778861 10.1016/0031-9384(95)02159-0

[53] Anderson, C., Von Keyserlingk, M., Lidfors, L. & Weary, D. Anticipatory behaviour in animals: A critical review. *Anim. Welf. ***29**, 231–238. 10.7120/09627286.29.3.231 (2020).

[54] Dantzer, R., Arnone, M. & Mormede, P. Effects of frustration on behaviour and plasma corticosteroid levels in pigs. *Physiol. Behav. ***24**, 1–4 (1980). 7189887 10.1016/0031-9384(80)90005-0

[55] Arnone, M. & Dantzer, R. Does frustration induce aggression in pigs?. *Appl. Animal Ethol. ***6**, 351–362. 10.1016/0304-3762(80)90135-2 (1980).

[56] Hill, H. M. M. et al. Cetacean responses to violation of expectation paradigm in a free-swim context. *Anim. Cogn. ***26**, 667–686. 10.1007/s10071-022-01704-7 (2023). 36333497 10.1007/s10071-022-01704-7

[57] Völter, C. J., Tomašić, A., Nipperdey, L. & Huber, L. Dogs’ expectations about occlusion events: From expectancy violation to exploration. *Proc. R. Soc. B. ***290**, 20230696. 10.1098/rspb.2023.0696 (2023). 37464755 10.1098/rspb.2023.0696 PMC10354481

[58] Špinka, M. Animal agency, animal awareness and animal welfare. *Anim. welf. ***28**, 11–20. 10.7120/09627286.28.1.011 (2019).

[59] Tallet, C. et al. Encoding of situations in the vocal repertoire of piglets (Sus scrofa): A comparison of discrete and graded classifications. *PLoS ONE ***8**, e71841. 10.1371/journal.pone.0071841 (2013). 23967251 10.1371/journal.pone.0071841 PMC3742501

[60] Marchant, J. N., Whittaker, X. & Broom, D. M. Vocalisations of the adult female domestic pig during a standard human approach test and their relationships with behavioural and heart rate measures. *Appl. Animal Behav. Sci. ***72**, 23–39. 10.1016/S0168-1591(00)00190-8 (2001). 10.1016/s0168-1591(00)00190-8 11259824

[61] Bensoussan, S., Tigeot, R., Meunier-Salaün, M.-C. & Tallet, C. Broadcasting human voice to piglets (Sus scrofa domestica) modifies their behavioural reaction to human presence in the home pen and in arena tests. *Appl. Animal Behav. Sci. ***225**, 104965. 10.1016/j.applanim.2020.104965 (2020).

[62] Murphy, E., Nordquist, R. E. & Van Der Staay, F. J. A review of behavioural methods to study emotion and mood in pigs, Sus scrofa. *Appl. Animal Behav. Sci. ***159**, 9–28. 10.1016/j.applanim.2014.08.002 (2014).

[63] Rault, J.-L. et al. Gentle abdominal stroking (‘belly rubbing’) of pigs by a human reduces EEG total power and increases EEG frequencies. *Behav. Brain Res. ***374**, 111892. 10.1016/j.bbr.2019.04.006 (2019). 30959126 10.1016/j.bbr.2019.04.006

[64] Friard, O. & Gamba, M. BORIS: A free, versatile open-source event-logging software for video/audio coding and live observations. *Methods Ecol. Evol. ***7**, 1325–1330. 10.1111/2041-210X.12584 (2016).

[65] Bateson, M. & Martin, P. *Measuring Behaviour: An Introductory Guide* (Cambridge University Press, 2021).

[66] Camerlink, I. & Ursinus, W. W. Tail postures and tail motion in pigs: A review. *Appl. Animal Behav. Sci. ***230**, 105079. 10.1016/j.applanim.2020.105079 (2020).

[67] Camerlink, I., Proßegger, C., Kubala, D., Galunder, K. & Rault, J.-L. Keeping littermates together instead of social mixing benefits pig social behaviour and growth post-weaning. *Appl. Animal Behav. Sci. ***235**, 105230. 10.1016/j.applanim.2021.105230 (2021).

[68] R Core Team. *R: A Language and Environment for Statistical Computing*. https://www.R-project.org/ (2024).

[69] Bates, D., Mächler, M., Bolker, B. & Walker, S. (2015) Fitting linear mixed-effects models using lme4. *J. Stat. Softw*. **67**, 1–48. 10.18637/jss.v067.i01.

[70] Brooks, M. E. *et al.* (2017) glmmTMB balances speed and flexibility among packages for zero-inflated generalized linear mixed modeling. *R J*. **9**, 378–400. 10.32614/RJ-2017-066.

[71] Field, A. Repeated-measures designs. *Discovering Statistics Using SPSS* 427–482. (2005).

[72] Quinn, G. P. & Keough, M. J. *Experimental Design and Data Analysis for Biologists* (Cambridge University Press, 2002).

[73] Fox, J. & Weisberg, S. *An R Companion to Applied Regression*. http://socserv.socsci.mcmaster.ca/jfox/Books/Companion (Sage, Thousand Oaks, 2019).

[74] Lenth, R. V. *Emmeans: Estimated Marginal Means, Aka Least-Squares Means*. https://CRAN.R-project.org/package=emmeans (2023).

[75] Forstmeier, W. & Schielzeth, H. Cryptic multiple hypotheses testing in linear models: Overestimated effect sizes and the winner’s curse. *Behav. Ecol. Sociobiol. ***65**, 47–55. 10.1007/s00265-010-1038-5 (2011). 21297852 10.1007/s00265-010-1038-5 PMC3015194

[76] Dobson, A. J. & Barnett, A. G. *An Introduction to Generalized Linear Models*, 4th Edn. 10.1201/9781315182780 (Chapman and Hall/CRC, 2018).

[77] Luke, S. G. Evaluating significance in linear mixed-effects models in R. *Behav. Res. ***49**, 1494–1502. 10.3758/s13428-016-0809-y (2017). 10.3758/s13428-016-0809-y 27620283

[78] Kuznetsova, A., Brockhoff, P. B. & Christensen, R. H. B. (2017) lmerTest package: Tests in linear mixed effects models. *J. Stat. Softw*. **82**, 1–26. 10.18637/jss.v082.i13.

[79] Therneau, T. M. & Grambsch, P. M. *Modeling Survival Data: Extending the Cox Model*. https://CRAN.R-project.org/package=survival (Springer, New York, 2000).

[80] Kassambara, A., Kosinski, M. & Biecek, P. *Survminer: Drawing Survival Curves Using ‘Ggplot2’*. https://CRAN.R-project.org/package=survminer (2021).

